# Meta-analysis of ultrasound-guided and traditional femoral artery puncture

**DOI:** 10.3389/fcvm.2023.1161834

**Published:** 2023-11-22

**Authors:** Jiazheng Li, Zhanjiang Cao, Tong Zhang, Keqiang Zhao, Junlai Zhao, Yu Yang, Chao Jiang, Zipeng Li, Rongrong Zhu, Weiwei Wu

**Affiliations:** Department of Vascular Surgery, Beijing Tsinghua Changgung Hospital, School of Clinical Medicine, Tsinghua University, Beijing, China

**Keywords:** femoral artery, ultrasound-guided puncture, traditional method, success on first attempt, hematoma

## Abstract

**Objective:**

To compare the ultrasound guidance and traditional methods in femoral artery puncture.

**Methods:**

We searched the databases to evaluate the rate of success on first attempt and the incidence of hematoma. The random effects model was used for performing a meta-analysis to estimate the odds ratio (ORs), mean difference (MD), and 95% confidence interval (CI).

**Results:**

A total of nine articles including 2,361 patients were included in this meta-analysis. The rate of success on first attempt were 79.6% (1,289/1,619) and 54.1% (883/1,644) in patients of the ultrasound group and traditional method group, respectively [OR = 3.14 (95% CI = 2.30–4.28), combined OR value *Z* = 7.23 (*P* < 0.00001)]. The rates of incidence of hematoma in the ultrasound group and traditional puncture group patients were 1.4% (16/1,168) and 3.8% (45/1,193), respectively (OR = 0.41, 95% CI = 0.17–1.00, *p* = 0.05).

**Conclusion:**

Ultrasound-guided femoral artery puncture has certain advantages compared with traditional puncture with regard to success on first attempt and the incidence of hematoma. Moreover, ultrasound-guided puncture reduces the incidence of hematoma in the retrograde puncture group patients.

## Introduction

Peripheral artery disease (PAD) affects approximately 200 million people worldwide (1), which is estimated to impact a large number of people (2). PAD associated with a 5-year significant morbidity is approximately 33.2% (3). If no timely treatment is provided to patients with PAD, the disease may progress to critical limb ischemia (CLI). The amputation rate of patients diagnosed with CLI within 1 year is 30% (4).

Traditionally, drugs and open surgery were used to treat diseases. In recent years, endovascular treatments have been increasingly adopted (5–7). Successful placement of the needle in the common femoral artery is an important surgical step, which is closely related to complications related to many vascular-access related complications (8). Improper positioning of the femoral artery puncture increases the risk of complications. Puncture below the bifurcation of the common femoral artery is more likely to lead to the formation of pseudoaneurysms (9–11). Conversely, puncture of the artery above the inguinal ligament is associated with a high incidence of retroperitoneal hemorrhage (12–15).

Traditionally, people used methods such as palpation of body surface markers and fluoroscopy to determine the location of the puncture. In recent years, ultrasound-guided puncture has been used increasingly because it provides the surgeon a more rapid access to the puncture site and causes fewer complications at the site (16). The purpose of this meta-analysis is to evaluate whether ultrasound guidance is associated with an increase in the rate of success on first attempt and a lower rate of hematoma.

## Materials and methods

### Literature search strategy and selection criteria

This report conforms to the Preferred Reporting Items for Systematic reviews and Meta-Analysis (PRISMA). We performed a comprehensive search of the CNKI, VIP, Wanfang, PubMed, Cochrane, and Embase databases for articles evaluating the efficacy of ultrasound guidance vs. traditional guidance of femoral arterial access. The last search was run on 28 September 2021. The following search strategy was used in articles published in the Chinese language: (chaosheng[Title/Abstract]) and ((gudongmai[Title/Abstract]) or (guqiandongmai[Title/Abstract]))) and ((toushi[Title/Abstract]) or (mangchuan[Title/Abstract])). The following search strategy was used in articles published in the English language. The search strategy was ((‘femoral artery’/exp OR (‘arteries, femoral’:ab,ti OR ‘artery, femoral’:ab,ti OR ‘femoral arteries’:ab,ti OR ‘common femoral artery’:ab,ti OR ‘arteries, common femoral’: ab, ti OR ‘artery, common femoral’:ab,ti OR ‘common femoral arteries’:ab,ti OR ‘femoral arteries, common’:ab,ti OR ‘femoral artery, common’:ab,ti)) AND (‘echography’/exp OR (‘ultrasonography, interventional’:ab,ti OR ultrasonography:ab,ti OR ‘diagnostic ultrasound’:ab,ti OR ‘diagnostic ultrasounds’:ab,ti OR ‘ultrasound, diagnostic’:ab,ti OR ‘ultrasounds, diagnostic’:ab,ti OR ‘ultrasound imaging’:ab,ti OR ‘imaging, ultrasound’:ab,ti OR ‘imagings, ultrasound’:ab,ti) OR (echotomography:ab,ti OR ‘ultrasonic imaging’:ab,ti OR ‘imaging, ultrasonic’:ab,ti OR ‘sonography, medical’:ab,ti OR ‘medical sonography’:ab,ti OR ‘ultrasonographic imaging’:ab,ti OR ‘imaging, ultrasonographic’:ab,ti OR ‘imagings, ultrasonographic’:ab,ti OR ultrasonographic:ab,ti) OR (echography:ab,ti OR ‘diagnosis, ultrasonic’:ab,ti OR ‘diagnoses, ultrasonic’:ab,ti OR ‘ultrasonic diagnoses’:ab,ti OR ‘ultrasonic diagnosis’:ab,ti OR ‘echotomography, computer’:ab,ti OR ‘computer echotomography’:ab,ti OR ‘tomography, ultrasonic’:ab,ti OR ‘ultrasonic tomography’:ab,ti) OR (‘ultrasound, interventional’:ab,ti OR ‘interventional ultrasound’:ab,ti OR ‘interventional ultrasonography’:ab,ti OR ‘ultrasonography, intravascular’:ab,ti OR ‘intravascular ultrasonography’:ab,ti)) AND (‘palpation’/exp OR ‘fluoroscopy’/exp OR (traditional:ab,ti OR anatomical:ab,ti OR fluoroscopic:ab,ti OR fluroscopy:ab,ti))) AND (‘health care quality’/exp OR (random:ab,ti OR ‘clinical trial’)).

**Figure 1:**
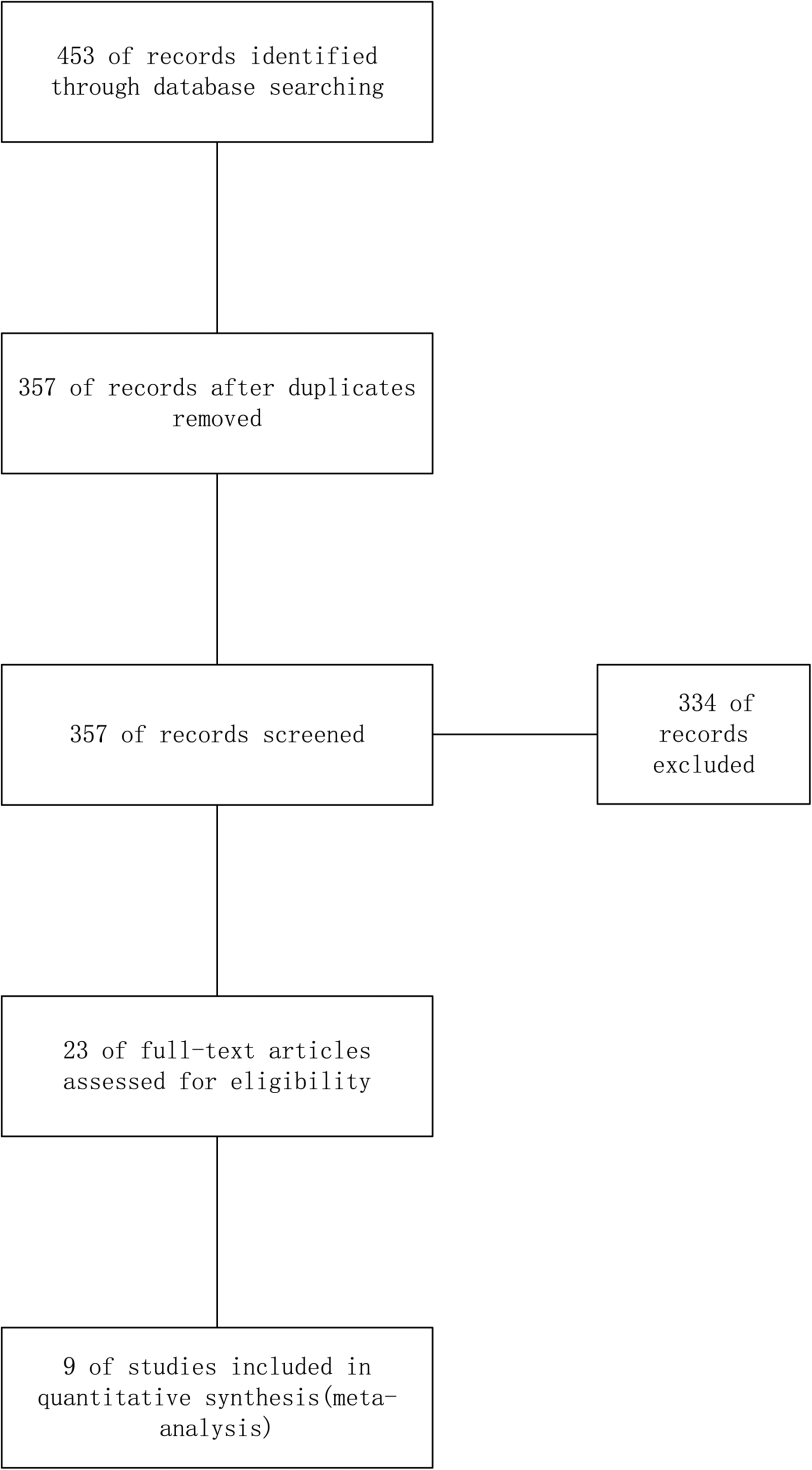
Flowchart of article screening and selection process.

Two authors (ZC and JL) independently assessed the eligibility of all retrieved studies. A third and a fourth author (WW and RZ) reviewed their findings. The investigators reached a consensus and the differences were resolved. The literature included in the meta-analysis was based on the following criteria: (1) randomized controlled trials; (2) the effect of ultrasound-guided femoral artery puncture was counted with the traditional femoral artery puncture as the control; (3) report on the rates of success on first attempt, or complications of the puncture. The study selected an initial search that identified 453 relevant articles, 430 of which were excluded after screening for titles or abstracts. After a careful reading of the remaining 23 articles, it was found that nine of them (3,313 patients) finally met the selection criteria and were, therefore, included in the current meta-analysis (Figure 1). The characteristics of all included studies are summarized in Table 1.

**Table 1:** Characteristics of the nine studies included in the meta-analysis.

Author	Year	Country	Study design	Intervention	Comparator	No. of patients^a^	Mean age (Y)^a^	Females^a^
Dudeck	2004	Germany	RCT	Ultrasound guidance	Traditional landmark technique in conjunction with guidance by palapation of the arterial pulse	56/56	—	24/18
Marquis-Gravel	2017	Canada	RCT	US-guided	anatomical landmark	64/65	65/67	16/18
Slattery	2014	Ireland	RCT	Ultrasound-guided	Fluoroscopy-assisted antegrade	53/47	68/66	15/16
Stone	2019	United States	RCT	Ultrasound	Fluoroscopic	319/316	65.4 ± 10.6/65.4 ± 11.6	157/159
Gedikoglu	2013	Turkey	RCT	Ultrasound Guidance	Traditional Palpation and Fluoroscopy Method	108/100	59.0 ± 15.2/59.5 ± 13.2	38/34
Siddik-Sayyid	2016	Lebanon	RCT	Ultrasound-guided	Palpation technique	53/53	37.9 ± 40.4 (month)/30.6 ± 25.7 (month)	20/24
Seto	2010	United States	RCT	US	Fluoroscopic	503/501	63.5 ± 12.4	132/135
Tremblay-Gravel	2015	Canada	RCT	Ultrasound-guided	Anatomical	40/40	—	—
Katircibasi	2018	Turkey	RCT	Ultrasound Guidance	Traditional palpation and fluoroscopy methods	449/490	60.3 ± 11.4/59.8 ± 10.6	216/233

^a^ The front is the intervention group and the back is the control group.

### Data extraction and quality assessment

Two authors (JL and ZC) independently extracted the following data from the included articles: first author, year of publication, study design, success rate of first puncture, success rate of total puncture, time of puncture, and complications. The seven main parts of the Cochrane Risk of Bias tool were used to evaluate the quality of all the articles: random sequence generation (selection bias), allocation concealment (selection bias), blinding of participants and personnel (performance bias), blinding of outcome assessment (detection bias), incomplete outcome data (attrition bias), selective reporting (reporting bias), and other biases.

### Heterogeneity and publication bias

Heterogeneity was assessed using the *I*^2^ statistic; values of <25%, 25%–50%, and >50% were considered low, moderate, and high heterogeneity, respectively. An *I*^2^ > 50% (*p* < 0.05) represented significant heterogeneity across the included studies. Potential publication bias was estimated by using the Begger's and Egger's tests.

### Outcome measures and statistical analysis

The rate of success on first attempt, total puncture success rate, puncture time, and complications in ultrasound puncture and traditional puncture were compared. The rate of success on first attempt: The number of patients with successful first common femoral artery puncture accounted for the proportion of the total number of patients in this group. Total success rate of puncture: the proportion of patients with common femoral artery cannulation after puncture in the group. Operation time: the recording of time from local anesthesia injection to vascular sheath implantation. Number of punctures: Each withdrawal of the needle is recorded as one time.

Because of the heterogeneity of the research, the random effects model was used to conduct a meta-analysis of the results. For continuous variables, if the mean and standard deviation were expressed in the same unit, they were combined into a mean difference with a 95% confidence interval. Odds ratio and 95% CI were used for categorical variables. Multivariate-adjusted ORs from cohort studies were pooled using generic inverse variance weighting. A subgroup analysis based on study design was conducted. Sensitivity analyses for the rate of success on first attempt were performed to test the reliability of the results by removing one study at a time and repeating the meta-analysis. A two-sided *p* < 0.05 indicated statistical significance. Analyses were performed using RevMan (version 5.3; Cochrane Information Management System; http://ims.cochrane.org/revman) and Stata software (version 14.0).

## Results

**Figure 2:**
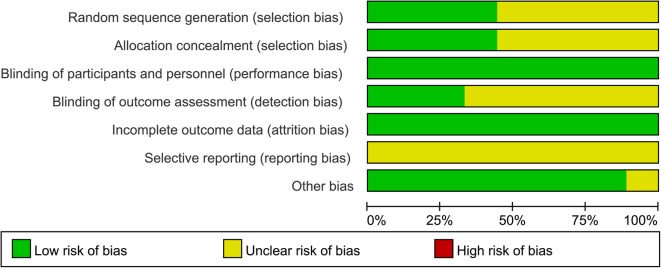
Risk of bias graph.

Nine articles involving 2,361 patients were included in this study (16–24). An insufficient blinding strategy in 9 RCTs increased the risk of bias. With regard to the blinding of Outcome Assessment, the difference in standards made the difference in results (Figure 2; Table 2).

**Table 2:** Risk of bias summary: review authors’ judgments about each risk of bias item for each included study.

	Random sequence generation (selection bias)	Allocation concealment (selection bias)	Blinding of participants and personnel (performance bias)	Blinding of outcome assessment (detection bias)	Incomplete outcome data (attrition bias)	Selective reporting (reporting bias)	Other biases
Dudeck et al. (17)	⋆	⋆	⋆	?	⋆	?	⋆
Gedikoglu et al. (18)	?	?	⋆	?	⋆	?	⋆
H. Seto et al. (19)	⋆	⋆	⋆	⋆	⋆	?	⋆
Katircibasi et al. (20)	?	?	⋆	?	⋆	?	⋆
M. Siddik-Sayyid et al. (21)	⋆	⋆	⋆	⋆	⋆	?	⋆
M. Slattery et al. (22)	?	?	⋆	?	⋆	?	⋆
Marquis-Gravel et al. (23)	?	?	⋆	?	⋆	?	⋆
Stone et al. (16)	⋆	⋆	⋆	⋆	⋆	?	⋆
Tremblay-Gravel et al. (24)	?	?	⋆	?	⋆	?	⋆

“⋆” means Low risk, “?” represents unclear risk, and “△” is high risk.

### The rate of success on first attempt

**Figure 3:**
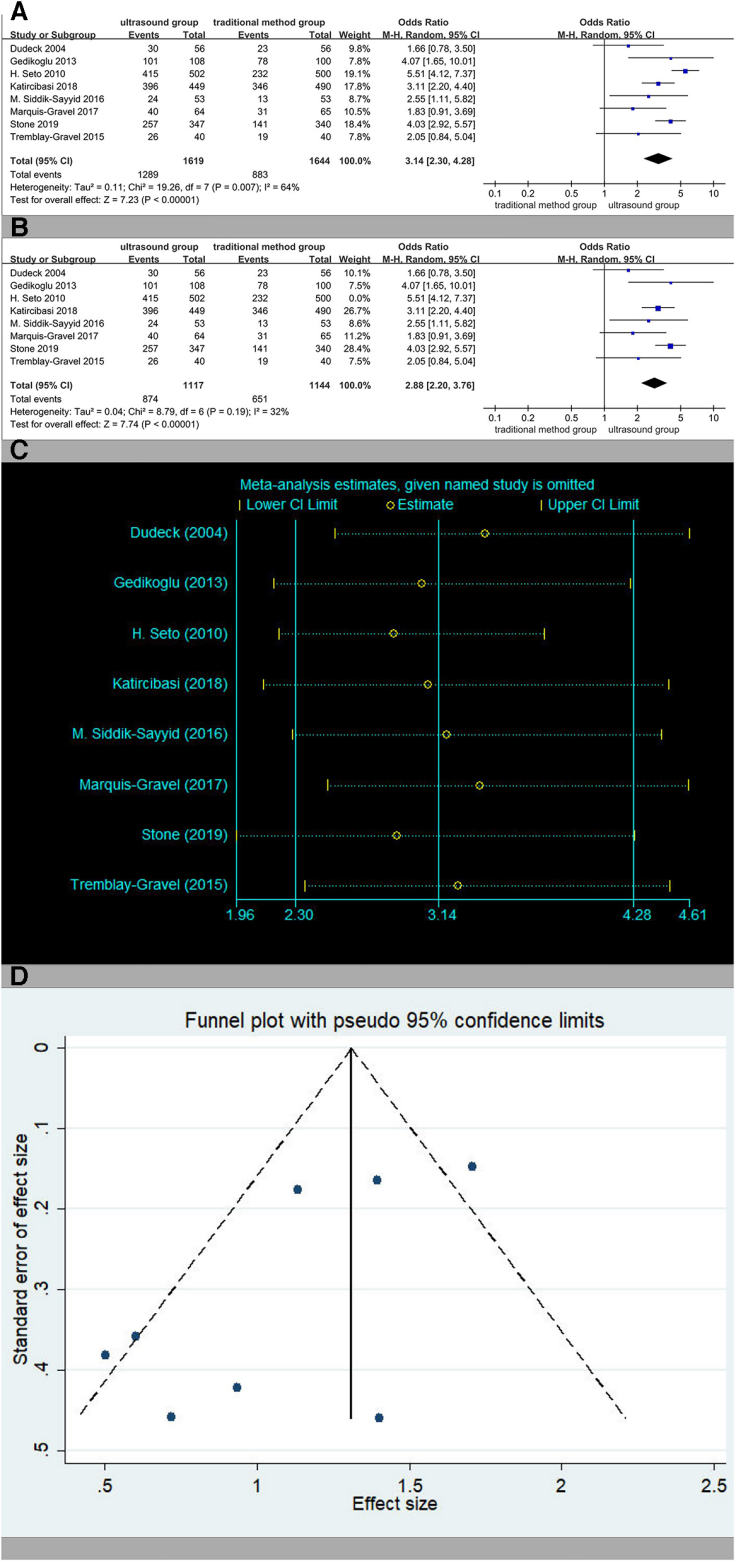
(**A**) The rate of success on first attempt. (**B**) The rate of success on first attempt except (19). (**C**) Sensitivity analysis of the rate of success on first attempt. (**D**) Funnel charts of the rate of success on first attempt.

Eight studies reported the rate of success on first attempt. The rates of success on first attempt in the ultrasound group patients were 79.6% (1,289/1,619) vs. 54.1% (883/1,644) in the traditional method group patients. The overall OR was 3.14 (95% CI 2.30–4.28), and the *Z*-score for the overall effect was *Z* = 7.23 (*P* < 0.00001), suggesting a significant difference between the two methods (Figure 3A). The heterogeneity in the studies reporting the first-pass success was high (*I*^2^ = 64%, *p* = 0.007). After the third study (19) was excluded, heterogeneity reduced significantly (Figure 3B), and the relevant reasons for this will be analyzed in the Discussion section. Sensitivity analysis showed that the estimates did not change significantly after other studies were excluded, implying that the result was relatively reliable (Figure 3C). The result of the Begger's test did not show significant publication bias (*p* = 0.386). After Egger's test, *p* was 0.044. The Trim and full Analysis showed that the result was relatively stable. Funnel charts were symmetrical (Figure 3D). In general, there was no publication bias for these inspection methods.

### Subgroup analyses based on the traditional method group

**Figure 4:**
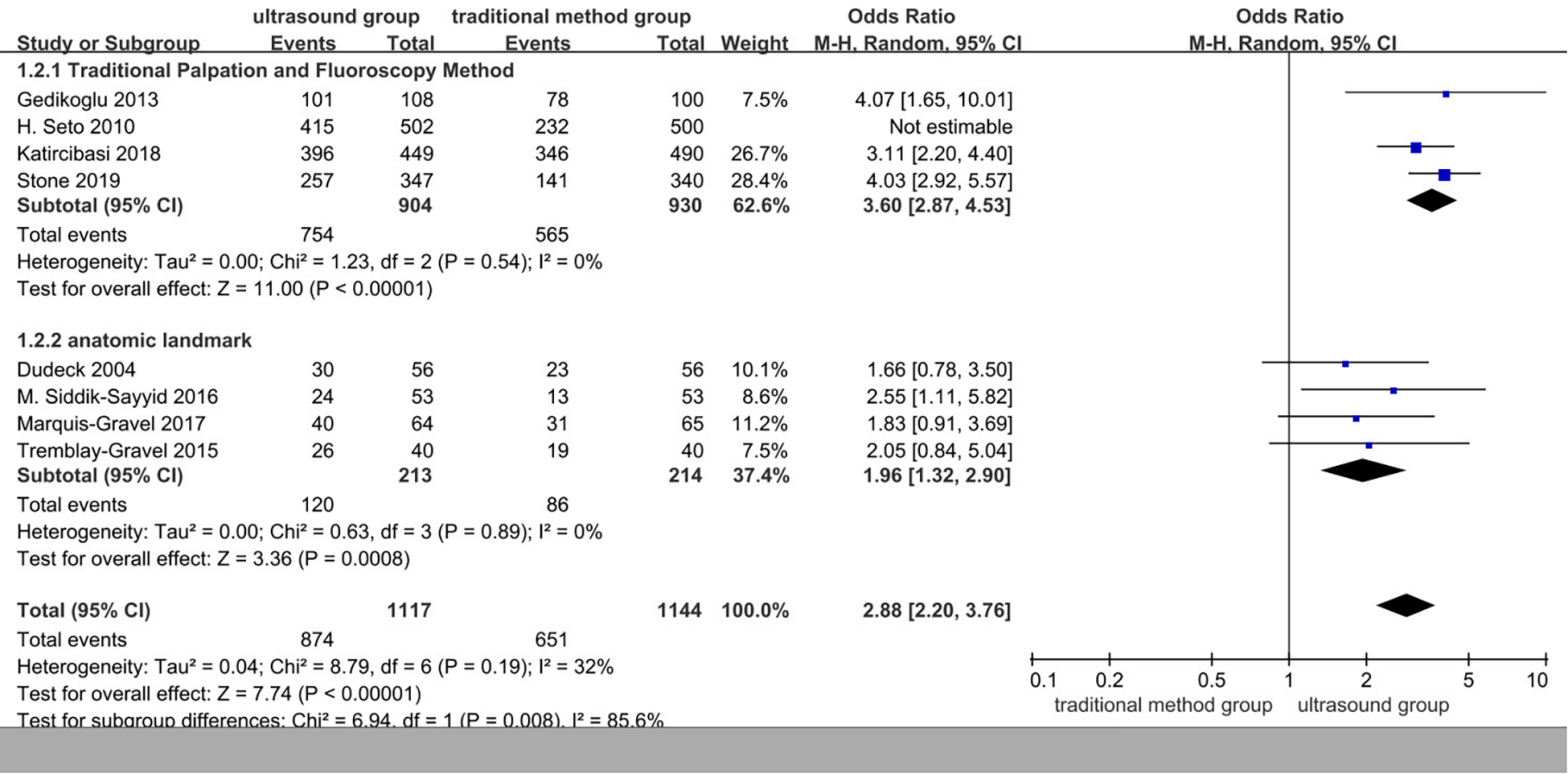
Subgroup of the first-pass success rate.

Subgroup analyses based on traditional methods vs. ultrasound are presented in Figure 4. Ultrasound was more effective than the traditional palpation and fluoroscopy method (OR 3.60, 95% CI 2.87–4.53, *p* < 0.00001). There were significant differences when compared with anatomic landmarks (OR 2.88, 95% CI 2.20–3.76, *p* < 0.00001), too. There was no significant heterogeneity.

### Total success rate

**Figure 5:**
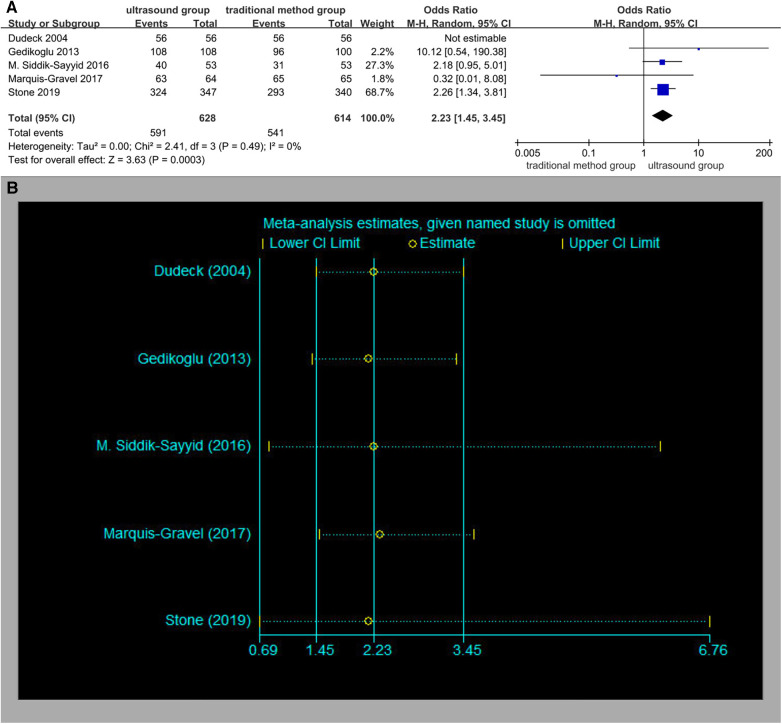
(**A**) Total success rate. (**B**) Sensitivity analysis of the total success rate.

Five studies reported the total success rate. The total success rates in the ultrasound group patients were 94.1% (591/628) vs. 88.1% (541/614) in the traditional method group patients. The overall OR was 2.23 (95% CI 1.45–3.45), and the *Z*-score for the overall effect was *Z* = 3.63 (*P* = 0.0003), suggesting a significant difference between the two methods (Figure 5A). There was no significant heterogeneity (*I*^2^ = 0%, *p* = 0.49). No significant publication bias was observed (Begger's test *p* = 0.734, Egger's test *p* = 0.902). The results of the sensitivity analysis are shown in Figure 5B.

### Rate of venipuncture

**Figure 6:**
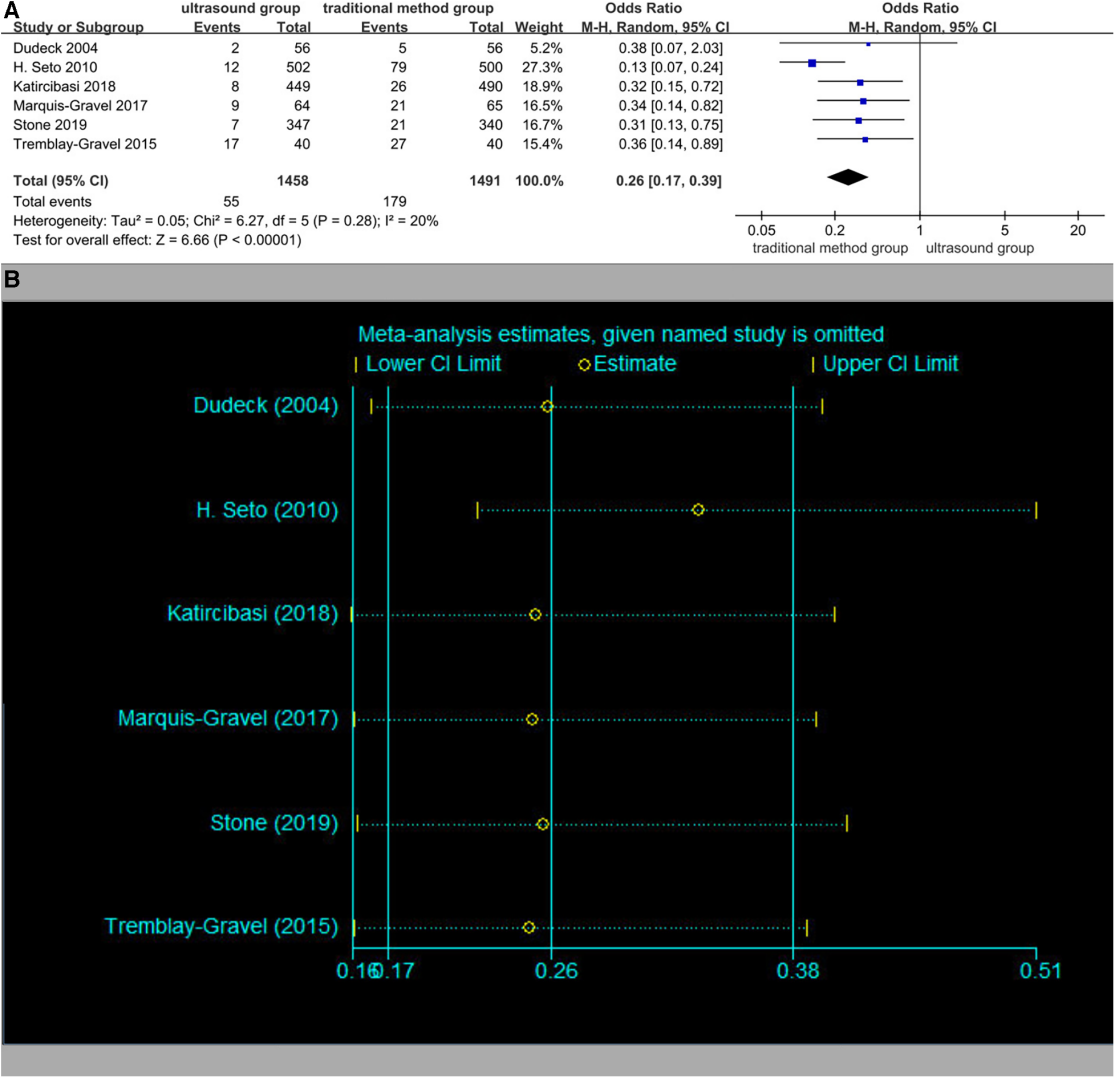
(**A**) Rate of venipuncture. (**B**) Sensitivity analysis of the rate of venipuncture.

Six studies reported the rate of venipuncture. The rates of venipuncture in the ultrasound group patients were 3.8% (55/1,458) vs. 12.0% (179/1,491) in the traditional method group patients. The overall OR was 0.26 (95% CI 0.17–0.39), and the *Z*-score for the overall effect was *Z* = 6.66 (*p* < 0.00001), suggesting a significant difference between the two methods (Figure 6A). The heterogeneity in the studies was low (*I*^2^ = 20%, *p* = 0.28). Sensitivity analysis showed that the estimates did not change significantly after each study was excluded, implying that those results were relatively reliable (Figure 6B). No significant publication bias existed in the rate of venipuncture (Begger's test *p* = 0.452, Egger's test *p* = 0.140).

### Rate of hematoma

**Figure 7:**
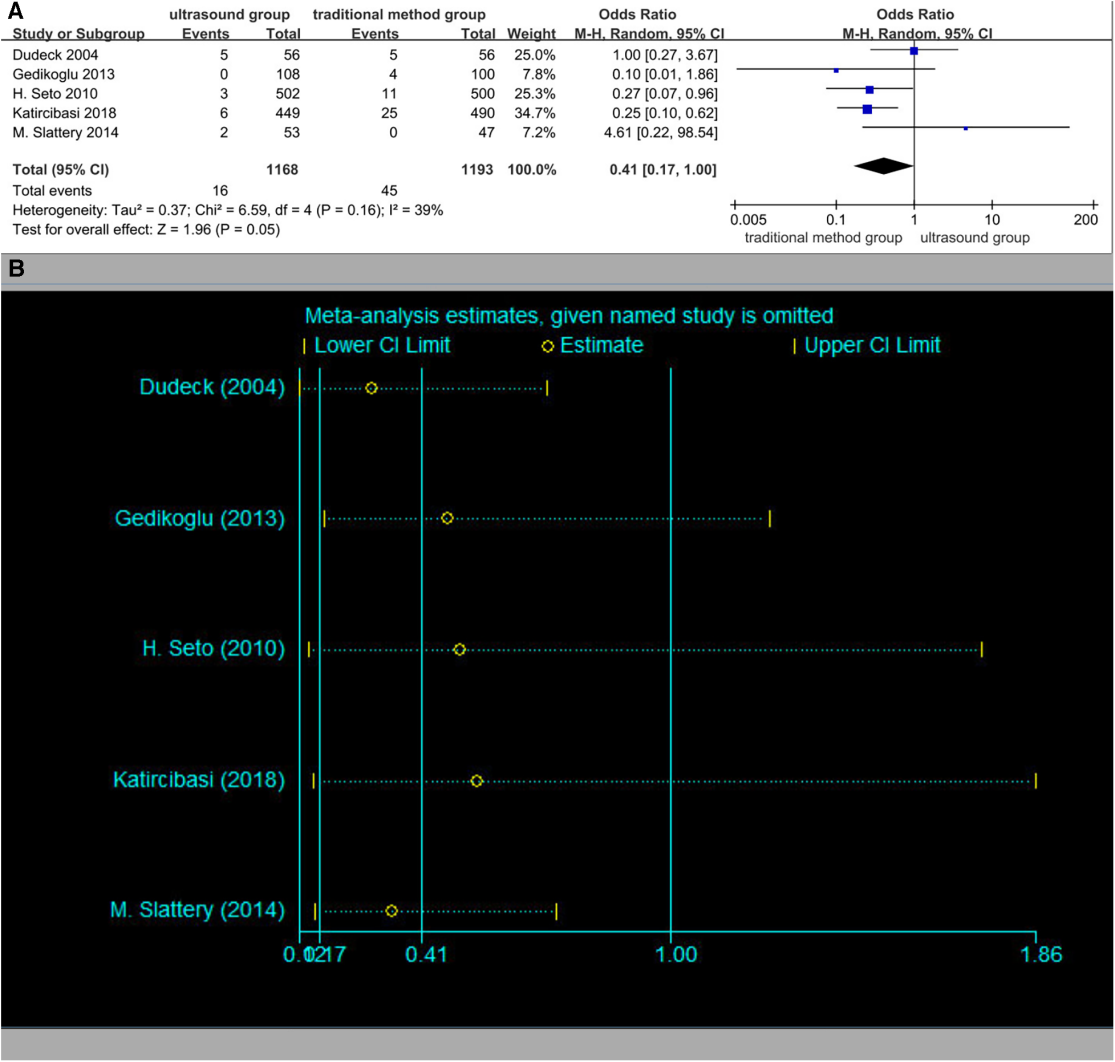
(**A**) Rate of hematoma. (**B**) Sensitivity analysis of the rate of hematoma.

Five studies reported the rate of hematoma (Figure 7A). The rates of hematoma in the ultrasound group patients were 1.4% (16/1,168) vs. 3.8% (45/1,193) in the traditional method group patients. The overall OR was 0.41 (95% CI 0.17–1.00). It was numerically less in the ultrasound group patients, although this was not statistically significant (*p* = 0.05). No significant publication bias was observed (Begger's test *p* = 0.462, Egger test *p* = 0.564). The results of the sensitivity analysis are shown in Figure 7B.

### Subgroup analysis based on antegrade or retrograde access

**Figure 8:**
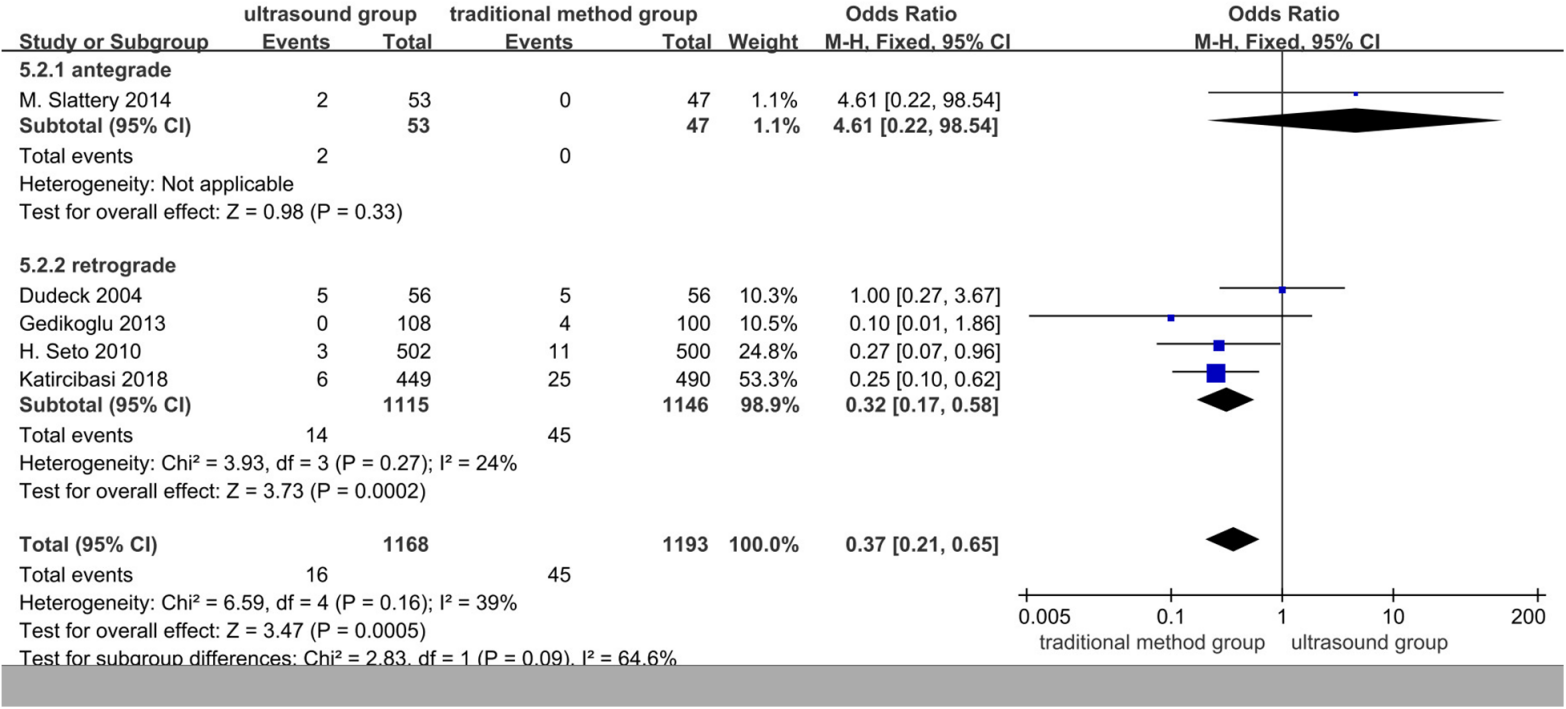
Subgroup of the rate of hematoma.

In the subgroup analyses based on antegrade or retrograde access, ultrasound was more effective than the traditional method (OR 0.37, 95% CI 0.21–0.65, *p* = 0.0005). When retrograde access was separated from antegrade access, there was less heterogeneity in the results (Figure 8).

### Other results

**Figure 9:**
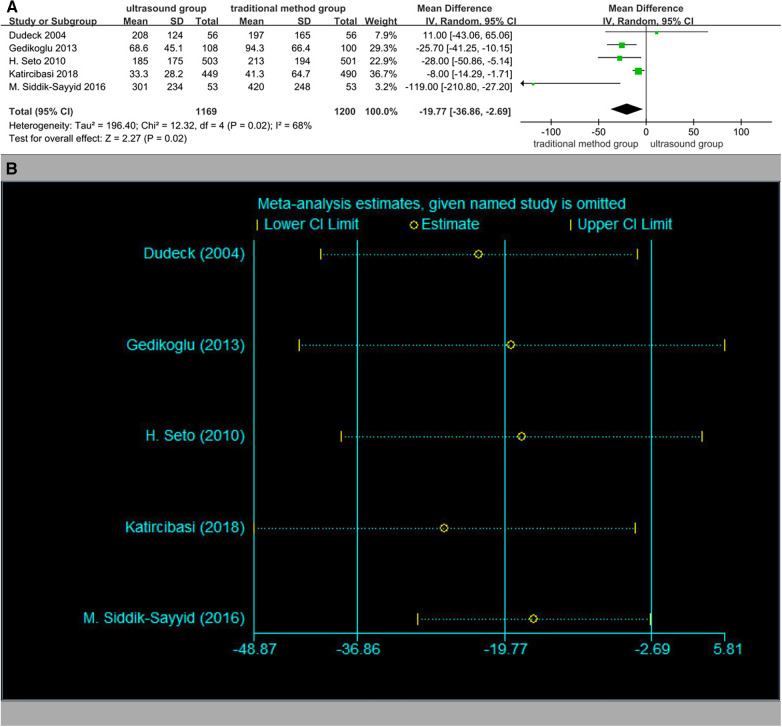
(**A**) Time to access the artery. (**B**) Sensitivity analysis of time to access the artery.

Time to access the artery was significantly less in the ultrasound group patients (Figure 9A). No significant publication bias was observed (Begger's test *p* = 0.806, Egger's test *p* = 0.489). The results of the sensitivity analysis are shown in Figure 9B.

**Figure 10:**
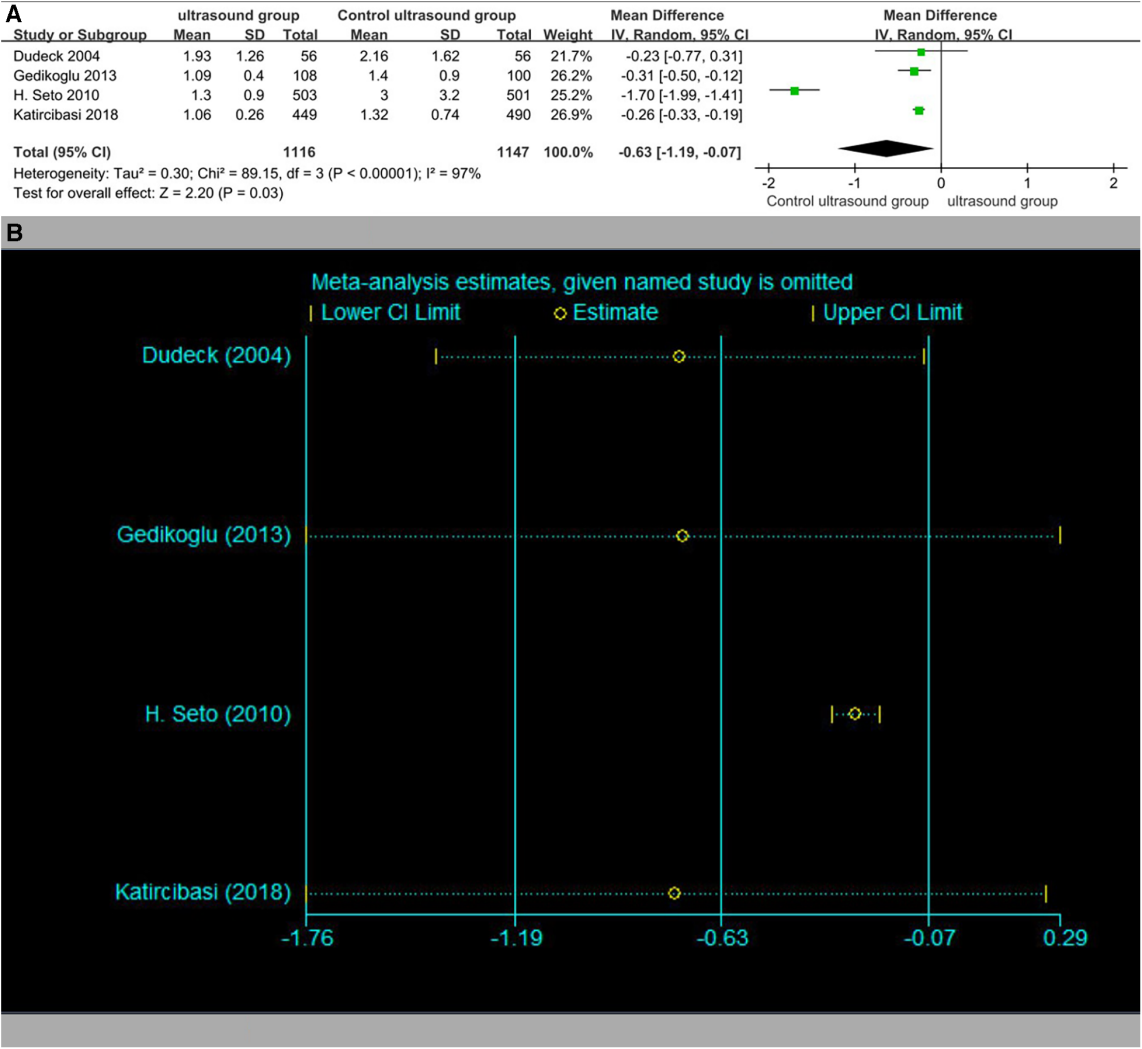
(**A**) The number of attempts. (**B**) Sensitivity analysis of the number of attempts.

In the ultrasound group patients, the number of attempts was obviously less (Figure 10A). No significant publication bias was observed (Begger's test *p* = 0.308, Egger's test *p* = 0.307). The results of the sensitivity analysis are shown in Figure 10B.

**Figure 11:**
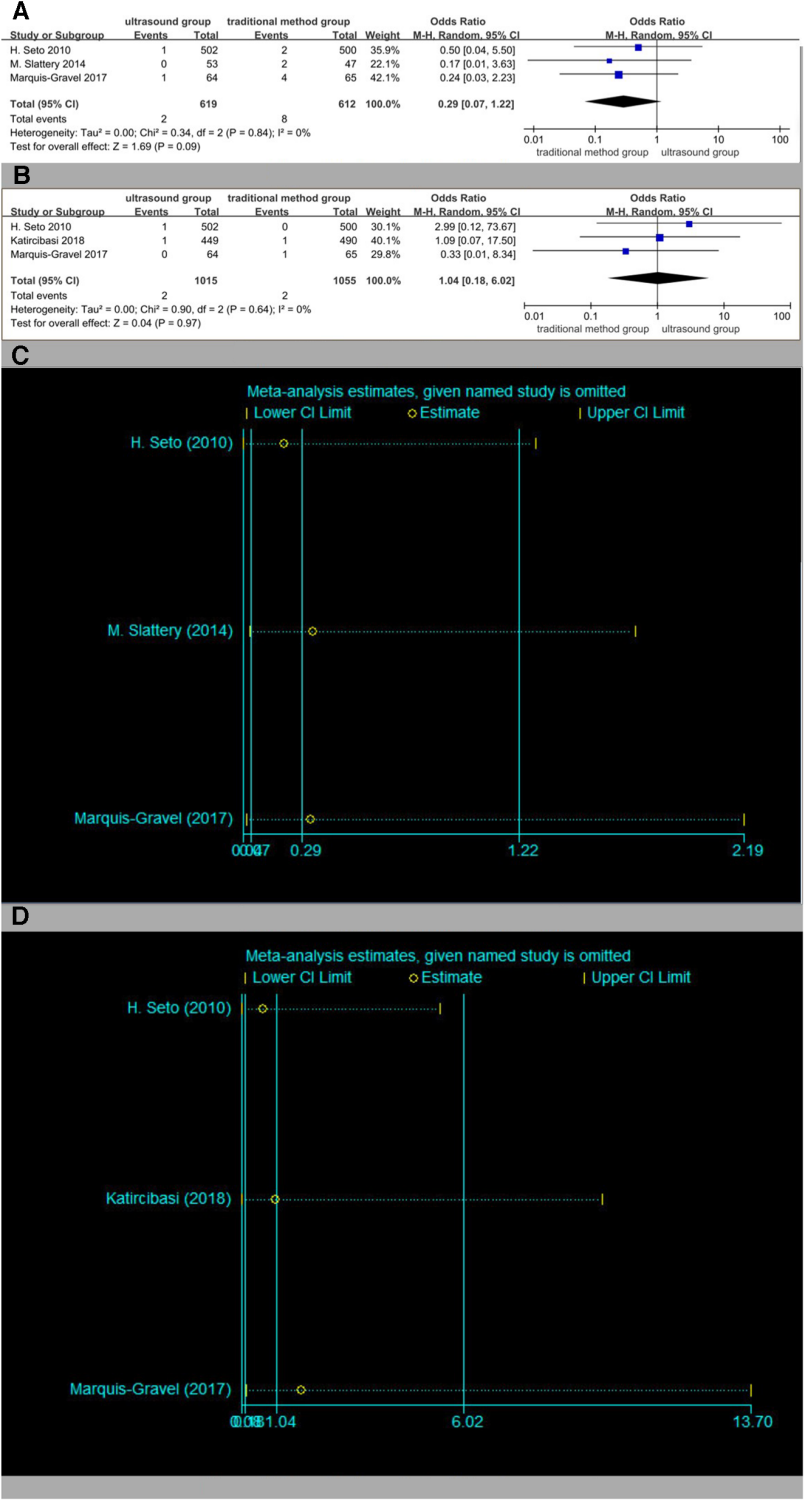
(**A**) Forest plot of bleeding. (**B**) Forest plot of pseudoaneurysm. (**C**) Sensitivity analysis of bleeding. (**D**) Sensitivity analysis of pseudoaneurysm.

Three studies were included in the analysis of the incidence of bleeding (Figure 11A) and the incidence of pseudoaneurysm (Figure 11B), respectively, with no statistical difference. The results of the sensitivity analysis are shown in Figures 11C,D.

## Discussion

The results showed that the rate of success on first attempt in the ultrasound group patients was 25% higher than that in the traditional puncture group patients, and the number of punctures also reduced by 0.6 times. The rate of hematoma caused by traditional puncture is a common problem when the pathway is established, and its incidence is about 1.7 times higher than that of ultrasound-guided puncture.

When analyzing the relevant data on the rate of success on first attempt, one of the articles (19) brought more heterogeneity. The article does not report on operator skill level by objective proficiency measures. The analysis shows that although operating proficiency has nothing to do with the total success rate, it is related to the operating time (19). The heterogeneity of the operator's proficiency may be the reason for the low success rate of ultrasound puncture for the first time in this article. The higher the number of punctures during the catheterization process, the more likely it is to damage the blood vessel wall and cause hematoma.

Hematoma is the most frequent local complication after puncture. The analysis in this article showed that the incidence of traditional punctures in patients with hematomas was slightly higher than that of ultrasound-guided puncture in patients with hematomas. A subgroup analysis of the incidence of postoperative hematoma based on antegrade or retrograde access can significantly reduce the heterogeneity of the incidence of hematoma in the subgroups. After subgroup analysis, it was found that the incidence of ultrasound-guided hematoma in patients who underwent retrograde puncture was significantly lower than that in those who underwent traditional puncture. The reasons for this include the higher rate of success on first attempt, less damage to blood vessels, and the easy-to-apply modified Seldinger method by which it is easier to puncture the anterior surface. A comparison of the previous fluoroscopy-guided antegrade (25) and ultrasound-guided antegrade (26) revealed that ultrasound-guided antegrade is less likely to cause hematoma, as it may be easier with ultrasound to successfully avoid puncturing the posterior wall of the artery and causing minor damage to the blood vessels (26). Therefore, it is considered that there is no significant difference in the rate of hematoma between the ultrasound-guided puncture method and the traditional method in the antegrade puncture group patients and the traditional puncture group patients, which may be related to the smaller sample size.

Although hematomas occurred in both patient groups, the ultrasound group patients had inguinal hematomas, and the traditional puncture group patients had retroperitoneal hematomas. Retroperitoneal hematomas may evolve into retroperitoneal hemorrhage. Retroperitoneal hemorrhage is often extremely dangerous (27). The occurrence of retroperitoneal hematoma is often associated with a higher puncture position (10, 11). In previous randomized controlled trials, the severity of hematoma was not distinguished, resulting in higher heterogeneity on the rate of hematoma. Therefore, if similar studies are to be carried out in the future, the type and size of hematomas should be further refined.

The difference between this study and previous studies is that the included randomized controlled trials have significantly increased, avoiding the previous situation where data from a single center accounted for the vast majority of patients (28). In this paper, the rate of success on first attempt is used as the primary endpoint because it is related to hematoma and to the catheterization time, which prolongs the overall time of the operation.

Because of the differences in the original documents, this study has limitations. The heterogeneity of the access time and the number of attempts are relatively high. There is no subgroup analysis of lesions in the vessel being punctured in the previous randomized controlled trials. The difficulty involved in puncture varies between patients with femoral artery diseases and those who need interventional treatment because of other vascular diseases. Bleeding is also a common complication, but the classification methods mentioned in each article are not uniform. They are often divided into major bleeding and non-bleeding, and therefore, a comprehensive grouping method can be considered (29).

Ultrasound guidance is effective for puncture in patients with conditions such as obesity, artery anatomical abnormalities, hypotension, and weak arterial pulsation (30). Ultrasound can also clearly determine the calcification of the blood vessel wall, and it is easier to puncture the healthy blood vessel area by using ultrasound than by using the anatomical positioning method, thus reducing the possibility of hematoma. The use of ultrasound adds part of the cost to patients, but if a local hematoma occurs, the required treatment cost is approximately 1,399$ (31). Ultrasound avoids greater risks with a small investment. Compared with fluoroscopy guidance, ultrasound-guided puncture does not require additional radiation (22). The disadvantage of ultrasound guidance is that its training cycle is long, and the puncture time of operators with different proficiency levels varies significantly (19), which is an obstacle to the popularization of ultrasound.

## Conclusion

Ultrasound-guided femoral artery puncture may have certain advantages compared with traditional puncture with regard to success on first attempt. In this study, we found that the possibility of hematoma occurring under ultrasound guidance was lower, but the difference was not obvious. Ultrasound was more effective in the retrograde group than in the traditional method group. The difference between the two methods necessitates a randomized controlled experiment with a larger sample size.
